# The Recent British Geneva Convention Act, 1911

**Published:** 1912-05-11

**Authors:** 


### THE ROYAL ARMY MEDICAL CORPS SECTION.

### The Recent British Geneva Convention Act, 1911.

The Red Cross activities in our midst, to which reference was made in a recent number of The Hospital, have made it more and more imperative that there should be amongst the public generally a clear understanding as to the exact meaning of the Red Cross badge. We quite admit that it is not imperative that everyone should know the story of its adoption, full of interest though it may be, or be aware of the fact that it is formed by reversing the Federal colours of Switzerland; but we do think that it should be a matter of general knowledge that it has been fixed on by all civilised nations as the recognised symbol for distinguishing the *personnel* and material of the ambulances and hospitals, both military and civilian, that have been detailed for use during a war. In view of its selection for this special object and of its being ear-marked, as it were, by the military authorities for this particular and important duty during hostilities, it follows that nothing justifies its use in civil hospitals, or in times of peace.

### Popular Delusions as to the Red Cross.

Such a procedure is quite indefensible and only serves to create confusion in the public mind, one result being that the idea becomes prevalent that in time of war anyone who chooses to tie a Red Cross badge on his or her arm is at liberty to give civilian assistance to the wounded on the field of battle, and that while doing so is not only exempt from capture, but has a perfect right to go where he or she likes. The fact is that the very reverse is the case, and that the emblem of the Red Cross on a white ground was adopted by an International Conference held at Geneva in 1864 with the object of preventing indiscriminate civilian aid in war time to an army in the field, as it was found that unauthorised outside assistance was an element of danger by opening up channels of espionage and pillage. To obviate these drawbacks the permission to wear the Red Cross badge was vested entirely in the hands of the military authorities, and it is now laid down that no badge has any value unless it bears the official stamp and a number. In short the Geneva Convention was established to ensure that every "benevolent neutral" who gives civilian help to the sick and wounded of an army in the field is doing so with the knowledge and approval of those in authority, and that he has agreed to carry on his duties in accordance with certain rules and regulations.

Of the abuse in time of war of the Red Cross, Longmore, in his "Manual of Ambulance Transport," gives several instances. Of these the most notable and the most flagrant was that of the Irish Ambulance, made up of about 300 Irishmen, who, in the Franco-German war of 1870, landed at Havre to the great astonishment of the inhabitants. Although their avowed object was to succour the sick and wounded, it was soon made evident that ambulance work was being made use of to facilitate their progress to the active area of hostilities, for soon afterwards they were found assisting in the defence of Châteaudun, having discarded their Red Cross badges and joined the combatant ranks of the French Army. Longmore also dwells on the manner in which the Red Cross is abused during peace, the very time when, as he says, every effort should be made to spread correct information as to the true meaning of an International Treaty, which is of such vast importance to humanity. In support of this he continues: "Hospital nurses also often adopt this badge, and some invalid attendants go so far as to introduce it in their advertisements. Surgical appliances and dressings, bandages, medicines, preserved food, aerated waters, soap, and all kinds of useful and useless things bear the same sign, and, in the event of war, the general ignorance which already prevails on the subject of the Geneva Convention would be emphasised by this wholesale and unauthorised adoption of a recognised International badge."

### The Remedy for the Abuse of the Cross.

From time to time efforts have been made by different Governments to afford some protection to the Red Cross badge. With this object the International Committee at Geneva offered prizes for the best essay on "The Abusive Employment of the Badge and Name of the Red Cross." The outcome of the suggestions offered was that the best and only satisfactory course was for all those Governments who have signed the Geneva Convention to agree to add to this document an additional article embodying their intention to enforce the protection of the badge. In this way it was left to each Government to pass such a law as would best suit the requirements and conditions of their respective countries. This has been done by several of the signatories to the Convention, and one of the latest to fall in with this course of procedure has been Great Britain, the Government having passed last August what is known as the Geneva Convention Act, 1911. Under it the use of the Red Cross is prohibited unless with the authority of the Army Council, and anyone convicted of employing it improperly will be liable on conviction to a fine not exceeding ten pounds, and to forfeit any goods upon or in connection with which the emblems or words were used. In the case of the proprietor of a trade mark registered before the passing of this Act, and containing any such emblem or words, he will be allowed to continue to use such trade mark for a further period of four years from the passing of the Act, but not subsequently. A clause has also been added extending the Act to the colonies and our foreign possessions, so that the colonies and foreign branches of the British Red Cross Society will benefit by its useful and very necessary provisions. With the adoption of this remedy against the indiscriminate use of the Red Cross badge and title good results should flow, and those who have the interests of the Red Cross work at heart must be satisfied at this further official recognition of it and at the protection thus afforded.

